# Transcriptional Evidence for the Role of Chronic Venlafaxine Treatment in Neurotrophic Signaling and Neuroplasticity Including also Glutatmatergic- and Insulin-Mediated Neuronal Processes

**DOI:** 10.1371/journal.pone.0113662

**Published:** 2014-11-25

**Authors:** Viola Tamási, Peter Petschner, Csaba Adori, Eszter Kirilly, Romeo D. Ando, Laszlo Tothfalusi, Gabriella Juhasz, Gyorgy Bagdy

**Affiliations:** 1 Department of Genetics, Cell- and Immunobiology, Semmelweis University, Budapest, Hungary; 2 Department of Pharmacodynamics, Semmelweis University, Budapest, Hungary; 3 MTA-SE Neuropsychopharmacology and Neurochemistry Research Group, Budapest, Hungary; 4 Neuroscience and Psychiatry Unit, University of Manchester, Manchester, United Kingdom; 5 Department of Neuroscience, Karoliska Institutet, Stockholm, Sweden; Peking University, China

## Abstract

**Objectives:**

Venlafaxine (VLX), a serotonine-noradrenaline reuptake inhibitor, is one of the most commonly used antidepressant drugs in clinical practice for the treatment of major depressive disorder (MDD). Despite being more potent than its predecessors, similarly to them, the therapeutical effect of VLX is visible only 3–4 weeks after the beginning of treatment. Furthermore, recent papers show that antidepressants, including also VLX, enhance the motor recovery after stroke even in non depressed persons. In the present, transcriptomic-based study we looked for changes in gene expressions after a long-term VLX administration.

**Methods:**

Osmotic minipumps were implanted subcutaneously into Dark Agouti rats providing a continuous (40 mg/kg/day) VLX delivery for three weeks. Frontal regions of the cerebral cortex were isolated and analyzed using Illumina bead arrays to detect genes showing significant chances in expression. Gene set enrichment analysis was performed to identify specific regulatory networks significantly affected by long term VLX treatment.

**Results:**

Chronic VLX administration may have an effect on neurotransmitter release via the regulation of genes involved in vesicular exocytosis and receptor endocytosis (such as *Kif* proteins, *Myo5a, Sv2b, Syn2* or *Synj2*). Simultaneously, VLX activated the expression of genes involved in neurotrophic signaling (*Ntrk2, Ntrk3*), glutamatergic transmission (*Gria3, Grin2b* and *Grin2a*), neuroplasticity (*Camk2g/b, Cd47*), synaptogenesis (*Epha5a, Gad2*) and cognitive processes (*Clstn2*). Interestingly, VLX increased the expression of genes involved in mitochondrial antioxidant activity (*Bcl2* and *Prdx1*). Additionally, VLX administration also modulated genes related to insulin signaling pathway (*Negr1, Ppp3r1, Slc2a4* and *Enpp1*), a mechanism that has recently been linked to neuroprotection, learning and memory.

**Conclusions:**

Our results strongly suggest that chronic VLX treatment improves functional reorganization and brain plasticity by influencing gene expression in regulatory networks of motor cortical areas. These results are consonant with the synaptic (network) hypothesis of depression and antidepressant-induced motor recovery after stroke.

## Introduction

Major depressive disorder is a highly complex disease characterized by several symptoms including depressed mood, diminished interest or tiredness and negative thoughts. [1]. According to earlier views, the main cause of MDD is the depletion of neurotransmitters serotonin (5-hydroxytryptamine, 5-HT) and noradrenaline (NA). This monoamine hypothesis was based on empirical observation about the mood-related effect of compounds used in non-mental disorders but capable to modify the levels of monoamines [2]. However, it turned out that the aetiology of MDD is more complex; besides of neurotransmitter depletions, the depressed brain also shows morphological abnormalities (changes in gray matter volume and neuronal organization), impairments in electrophysiological activity as well as in receptor pharmacology [3]. In this context, mood disorders are thought to be resulted from an inability of the neuronal networks that guide mood-related behaviour adjusting to inputs from the external world optimally (network hypothesis) [4], [5].

Despite extensive research, response of depressed patients to the currently available pharmacological therapies is rather unpredictable and varies widely, namely, 30–40% of patients do not respond. Yet, one of the best tolerated antidepressant drug used in MDD is venlafaxine (VLX), which seems to be more advantageous compared to selective serotonin reuptake inhibitors both in terms of remission rates and economical costs [6]. VLX is characterized as a serotonin and noradrenaline reuptake inhibitor (SNRI). To a lesser degree, it also blocks dopamine reuptake and exhibits a mild β-receptor antagonistic activity [7]. The acutely enhanced availability of extracellular 5-HT and NA is, however, likely not responsible for the antidepressant action of the drug directly since VLX exerts its positive effects on mood only after a few weeks (three and four weeks in male and female individuals, respectively) [6], [8]. Rather, gradual adaptation to the enhanced monoaminergic neurotransmission; e.g. desensitization of counteracting mechanisms [9], establishment of new neuronal connections and changes in synaptic plasticity as well as information processing, may all be responsible for the treatment effect of VLX [10].

As it was shown by human *in vivo* imaging- or *post mortem* studies for several brain areas, including regions of frontal, prefrontal and cingulate cortices, limbic system, hippocampus, striatum, amygdala and thalamus that they can mediate the diverse symptoms of depression [11]. Most of the studies dealing with depression focus on the limbic system and the prefrontal cortex, since these brain areas are critically involved in emotion processing and executive control. However, to the best of our knowledge, only few papers are available that investigates the role of other brain regions, such as frontal cortex (FC, motor cortical areas) in depression and even those studies do not examine the effects of VLX after a chronic administration which would have a substantial clinical relevance.

A recent study found a strong association between depressed mood and altered locomotor patterns (decreased locomotor activity and intermittent periods of low activity) [12]. Also, the co-morbidity of depression with disorders that affect the FC [e.g. frontal lobe atrophy [13] or multiple sclerosis [14]], is well established.

On the other side, earlier preliminary studies and a recent meta-analysis confirmed that selective serotonin reuptake inhibitors improve motor recovery after stroke, even in people who were actually not depressed [15], [16]. In a randomized, double blind, crossover study 1 week treatment with VLX significantly improved finger-tapping rate, a motor task compared to placebo [17]. This improvement showed positive correlation with the activation of sensory and motor cortical areas caused by the drug [17].

The aim of the present study is to investigate the potential changes in gene expressions of the frontal cortex following chronic VLX administration of Dark Agouti rats. In addition, by performing gene set enrichment analysis, we also studied the molecular alterations in regulatory networks, which may help to understand how gene expression changes lead to the clinical action of VLX.

## Methods

### Animals and Drugs

In this study, 20 male Dark Agouti rats (Harlan, Olac Ltd, Shaw's Farm, Blackthorn, Bicester, Oxon, UK, aged: 8 weeks, weighing 158±4 g [mean ± S.E.M.) were used. The animal experiments and housing conditions were carried out in accordance with the European Community Council Directive of 24 November 1986 (86/609/EEC), as well as the National Institutes of Health Principles of Laboratory Animal Care (NIH Publication 85-23, revised 1985) and special national laws (the Hungarian Governmental Regulation on animal studies, 31 December 1998 Act). The experiments were approved by the National Scientific Ethical Committee on Animal Experimentation and permitted by the Food Chain Safety and Animal Health Directorate of the Central Agricultural Office, Hungary (permission number: 22.1/3152/001/2007).

Prior to implantation, Alzet 2001 osmotic minipumps (Durect Corp., CA, USA) were filled with VLX dissolved in 0.9% NaCl solution.

### Drug Administration and Experimental Design

The animals were randomly divided into two groups according to the treatments. In VLX treated group, Alzet osmotic minipumps were implanted subcutaneously under the back skin of the animals, delivering 40 mg/kg VLX each day. The control group underwent sham surgery without the implantation of osmotic minipump. All surgeries were performed under halothane anaesthesia, and all efforts were made to minimize suffering of the animals. Following surgery, animals were returned to their home cages and were kept there until further processes. Food and water were available *ad libitum* for each animal. During surgical procedures one animal died, thus, altogether 19 animals were used in the experiments.

### RNA Extraction and Sample Preparation

Three weeks after the first osmotic minipump insertion rats were sacrificed quickly by decapitation. The brains were removed; approximately 2 mm thick coronal sections were cut and the FC regions (M1, M1 and Fr2), were dissected out according to Paxinos and Watson [18], (between approximately bregma +1.7 and +3.7) and stored at −80°C. The samples were homogenized with 1 ml TRIzol reagent and RNA was isolated as it was described before [19]. The pellets were dissolved in 20 µl diethylpyrocarbonate-treated-dH_2_O (DEPC-dH_2_O) and the samples stored at −80°C until further processing. To determine the quality of the samples, 1–2 µl were used for optical density (OD, 260/230 and 260/280 ratios) measurements. The OD ratios were determined for all samples and randomly repeated to evaluate the reliability of the measurements (no significant difference was observed, data not shown). Samples with the lowest RNA concentrations were excluded from further analysis and thus both VLX and control groups consisted of 8 animals. From these samples, two-two randomly selected samples were pooled. From VLX treated and vehicle-treated pools microarray experiments were performed by Service XS (Leiden, Netherlands) on the Illumina platform (RatRef-12 v1 Beadarray Expression Chip, San Diego, CA, USA).

### Data Analysis

Raw microarray data were processed with beadarray [20], preprocessCore [21] and puma [22] Bioconductor [23] packages for R [24] as described in [25]. Briefly, backgroundCorrect method, used in the beadarray package, was set to “minimum”, and “log = TRUE; n = 10” variables were used for createbeadsummaryData method. The normalization was performed by the ‘quintile method’ in the preprocessCore package. Additionally, pumaComb, pumaDE, and write.rslts functions with default settings were applied. Changes were considered statistically significant when the MinPplr was below 0.005.

Heat map visualization of the differences in gene expression was done using Multiexperiment Viewer Tool [26], [27]. Genes with similar expression patterns are grouped together with hierarchical clustering (Euclidean distance, average linkage, predicted genes and locus predictions were excluded) [28].To provide an even more wide-scale analysis of the possible pathways involved in VLX effects (e.g. neuropathic pain and migraine related pathways), we used textmining methods in NCBI's medical databases. The underlying principle for this extended method was the well-known fact that VLX has positive effects in both of the latter conditions (e.g. see [29] and [30]). To estimate the bibliometric relations between genes and neuropathic pain we counted the hits of the Pubmed queries “<gene name> AND (pain OR neuropath* OR nocicept* OR migraine)” [31]). After the identification of the possible genes, we used individually written R scripts [32] with the genome wide annotation database for Rattus Norvegicus [33] and the Gene Ontology (GO) annotation database [34] from Bioconductor [23] to reversely map these genes in the GO hierarchy. The resulting GO terms were then filtered to contain more than 15 and less than 500 genes for valid statistical analysis [35] and these terms together with the MSigDB C5 GO terms (corresponding to the same criterion) formed the input of the Gene Set Enrichment Analysis (GSEA).

GSEA was performed using the version 3.1 from the Broad Institute at MIT (http://www.broadinstitute.org/gsea) [36]. Gene identifiers used in the array dataset and gene sets were gene symbols. The data set had 22523 features (Illumina probes), which were collapsed to gene symbols (the median expression value was used for the probe set). T-test was used as the metrics for ranking genes and “gene set” algorithm was chosen as the permutation type since the sample size was less than 7 in this study. 1000 permutations were used to calculate p-value with the seed of permutation set to 149. All other parameters were set to default.

A normalized enrichment score (NES) was calculated for each gene set to represent the degree in which it was enriched in one phenotype. The nominal p-value and the FDR corresponding to each NES were calculated. A NES with a nominal p-value<0.05, FDR<0.25 were considered statistically significant. Network visualization and analysis using enrichment results was done using Cytoscape 2.8.3. and its plug in “Enrichment Analyzer” with the following cut-offs: similarity coefficient cut-off 0.1, p-value cut-off 0.05 and FDR cut-off 0.25 [37], [38].

### PCR Validation

We have validated 19 RNA products from the original pooled samples with real-time polymerase chain reaction (PCR) on Fluidigm GEx array (San Francisco, CA, USA) using Taqman Gene Expression assays for the appropriate RNAs obtained from Applied Biosystems (Carlsbad, CA, USA). Each sample was used in duplicate following quality control measurements. The validation experiment was performed by Service XS (Leiden, Netherlands). Upon arrival of the normalized results, manually written R scripts using the cor.test function with default settings were used for the comparison between microarray and PCR data. The Pearson correlation coefficients were 0.421 and 0.438, while the p-values were 0.0085 and 0.006 for the 200 ng and 500 ng samples, respectively (the Spearman correlation coefficients were 0.552 and 0.572 for the 200 ng and 500 ng samples, respectively; while respective p-values were below 0.001).

### Availability of supporting data

The data supporting the results of this publication have been deposited in NCBI's Gene Expression Omnibus [39] and are accessible through GEO Series accession number GSE47541 (http://www.ncbi.nlm.nih.gov/geo/query/acc.cgi?acc=GSE47541).

## Results

### Profiling mRNA expression after treatment with VLX

**Figure 1:**
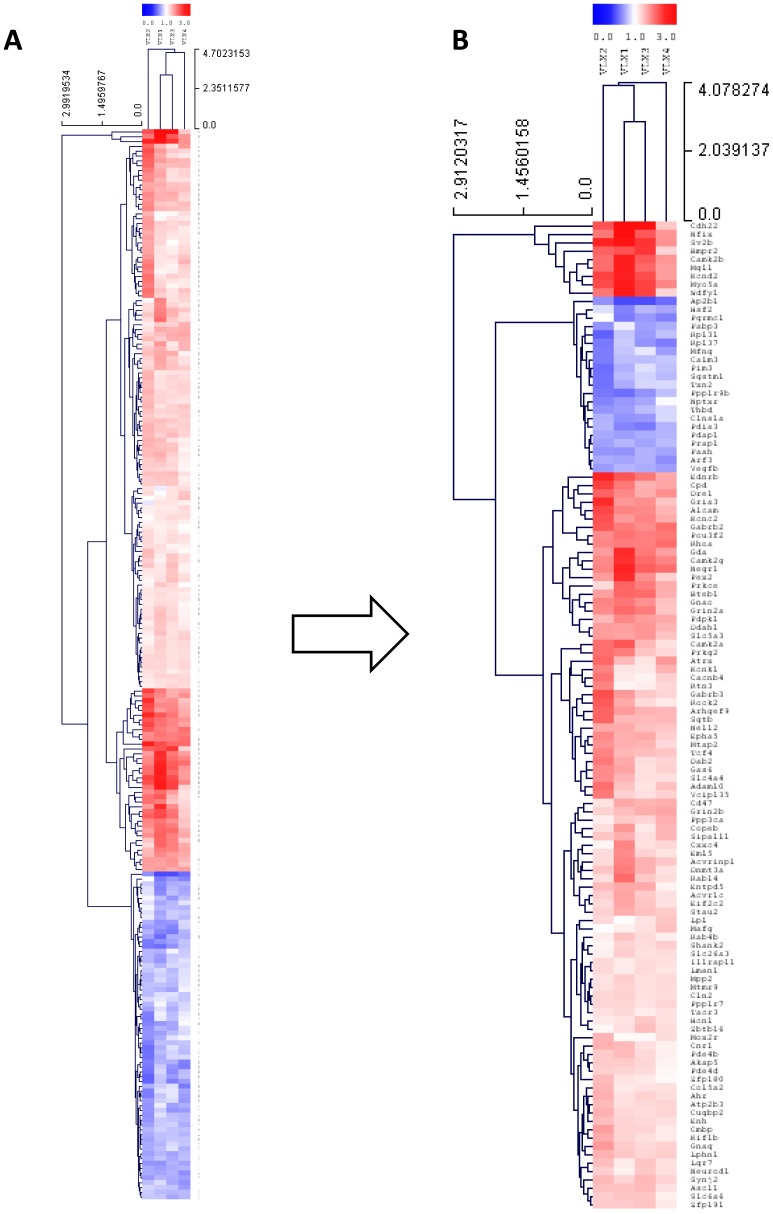
Significantly changed genes after three-week-long venlafaxine administration in the frontal cortex of rats. (A) All significantly changed genes clustered to a heat map (Euclidean distance, average linkage, p<0.005, red; upregulated, blue; downregulated). (B) Genes modulated more than 1.2 or less than 0.8 are clustered. Heat maps were produced using simultaneous clustering of rows and columns of the data matrix using average linkage algorithm and a Euclidean distance metric. The mRNA clustering tree is shown on the left and the sample clustering tree is shown on the top. The colour scale shown at the right illustrates the fold change of the indicated mRNA compared to control: red denotes fold change>1 and blue denotes fold change <1 (Minimum probability of positive log ratio <0.005).

Comparison of the gene expression profiles showed 222 genes expressed differentially in the VLX treated group compared to the saline control (minimum probability of positive log ratio (MinPplr) <0.005) (Figure 1/A.). From these, 118 defined genes (gene activity of 97 genes was up- and 21 genes was downregulated) showed changes higher than 1.2- or lower than 0.8-fold alteration (Figure 1/B.).

### Network analysis

**Figure 2:**
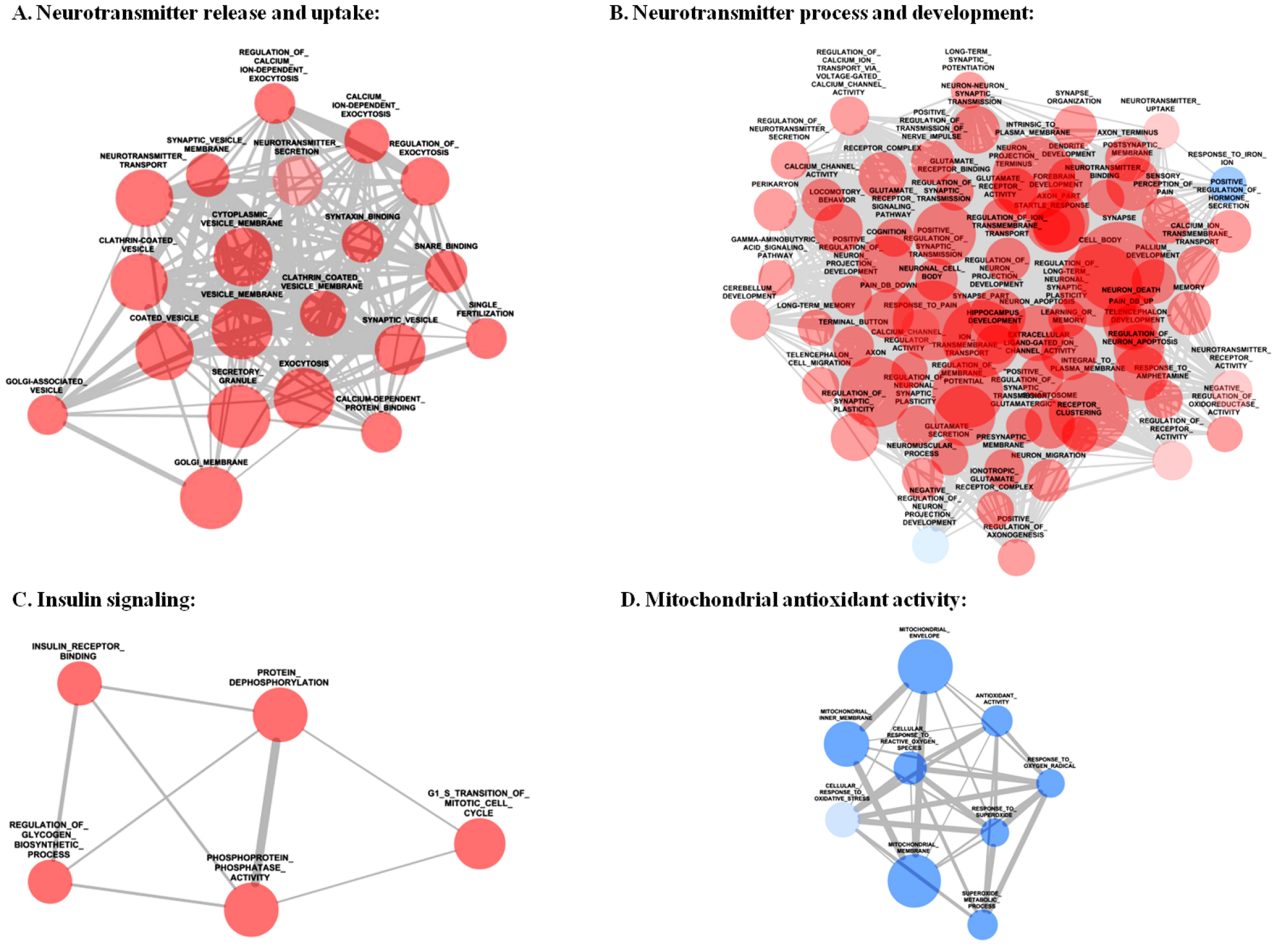
Network analyses of significantly enriched gene sets after three-week-long venlafaxine administration in rats. Nodes represent gene ontology (GO) terms significantly changed in gene set enrichment analysis (p<0.05, false discovery rate <0.25). Blue and red circles represent down-and upregulation of the associated GO terms, respectively. The size of the nodes is proportional with the number of genes in the GO term and the thickness of grey edges represents the number of common genes between two GO terms (A) Neurotransmitter release and uptake, (B) Neuronal processes and development, (C) Insulin signaling, (D) Mitochondrial antioxidant activity.

To analyze the functional outcome, namely, to identify activated or depressed gene clusters following long-term VLX treatment in the FC, we chose pathway-centric statistical approach, GSEA, in which functionally interacting genes were analyzed. Beside well-described, canonical GO-pathways derived from MSigDB, we intended to increase the chance to find yet unidentified networks in the effects of chronic VLX-treatment. For this purpose, we focused also on pathways found with text mining as described in methods section. To reduce spurious findings, we chose a restrictive false discovery rate cut off <0.25 for selecting enriched gene sets. The results of GSEA were visualized in Cytoscape and with the mentioned criteria, 525 gene sets (nodes) and 29259 interactions (edges) were found. To interpret the results, the interactome was clustered with spectral clustering [40] in Cytoscape to smaller subnetworks (Figure 2A–D).

#### Neurotransmitter release and uptake

This cluster represents an interaction between 20 upregulated gene sets with a nominal enrichment score (NES) range between 1.39–1.96. Functions of these genes were related to both neurotransmitter transport and secretion, synaptic endo- or exocytosis as well as regulation of these processes. The top five gene sets, with the highest NES were as follows: ‘regulation of exocytosis’ (NES = 1.96), ‘exocytosis’ (NES = 1.85), ‘synaptic vesicle’ (NES = 1.77), ‘synaptic vesicle membrane’ (NES = 1.71) and ‘regulation of calcium ion dependent exocytosis’ (NES = 1.7) (Figure 2A).

#### Neuronal processes and development

This cluster reveals interaction between 76 gene sets representing general neuronal processes and functions, such as ‘synaptic plasticity’, ‘synaptosome’, ‘neuron migration’, ‘neuron death’, glutamate signaling’, ‘memory’, learning’ and ‘cognition’ with NES range 2.36-(−1.72). The top five gene sets, with the highest NES were as follows: ‘terminal button’ (NES = 2.36), ‘pallium development’ (NES = 2.16), ‘regulation of long-term neuronal synaptic plasticity’ (NES = 2.15), ‘telenchephalon development’ (NES = 2.07) and ‘synapse part’ (NES = 2.05). There were two downregulated gene sets: ‘response to iron ion’ (NES = −1.72) and ‘negative regulation of neuronal projection development’ (NES = −1.45). The latter one shows downregulation of inhibitory genes, which practically means the facilitation of neuronal projection development (Figure 2B).

#### Insulin signalling

The subnetwork is composed of 5 upregulated gene sets with NES values ranged between 1.2–1.9. Four from these five networks, such as ‘insulin receptor binding’ (NES = 1.55), ‘phosphoprotein phosphatase activity’ (NES = 1.28), ‘protein dephosphorylation’ (NES = 1.26) and ‘regulation of glycogen biosynthetic process’ (NES = 1.89) could be directly linked to insulin signaling (Figure 2C).

#### Mitochondrial antioxidant activity

This small interactome is made from 9 downregulated gene sets, which are related to superoxide metabolism. From these, the following five gene sets exhibited the highest level of downregulation: (i) ‘superoxide metabolic process’ (NES = −1.82), (ii) ‘response to oxygene radical’ (NES = −1.78), (iii) ‘mitochondrial inner membrane’ (NES = −1.77), (iv) ‘response to superoxide’ (NES = −1.73) and (v) ‘mitochondrial envelope’ (NES = −1.53) (Figure 2D).

### Genes with significantly altered expression and established or suspected involvement in the pathomechanism of depression

#### (i) Genes with described role in depression or in the molecular mechanism of antidepressant therapy

**Figure 3:**
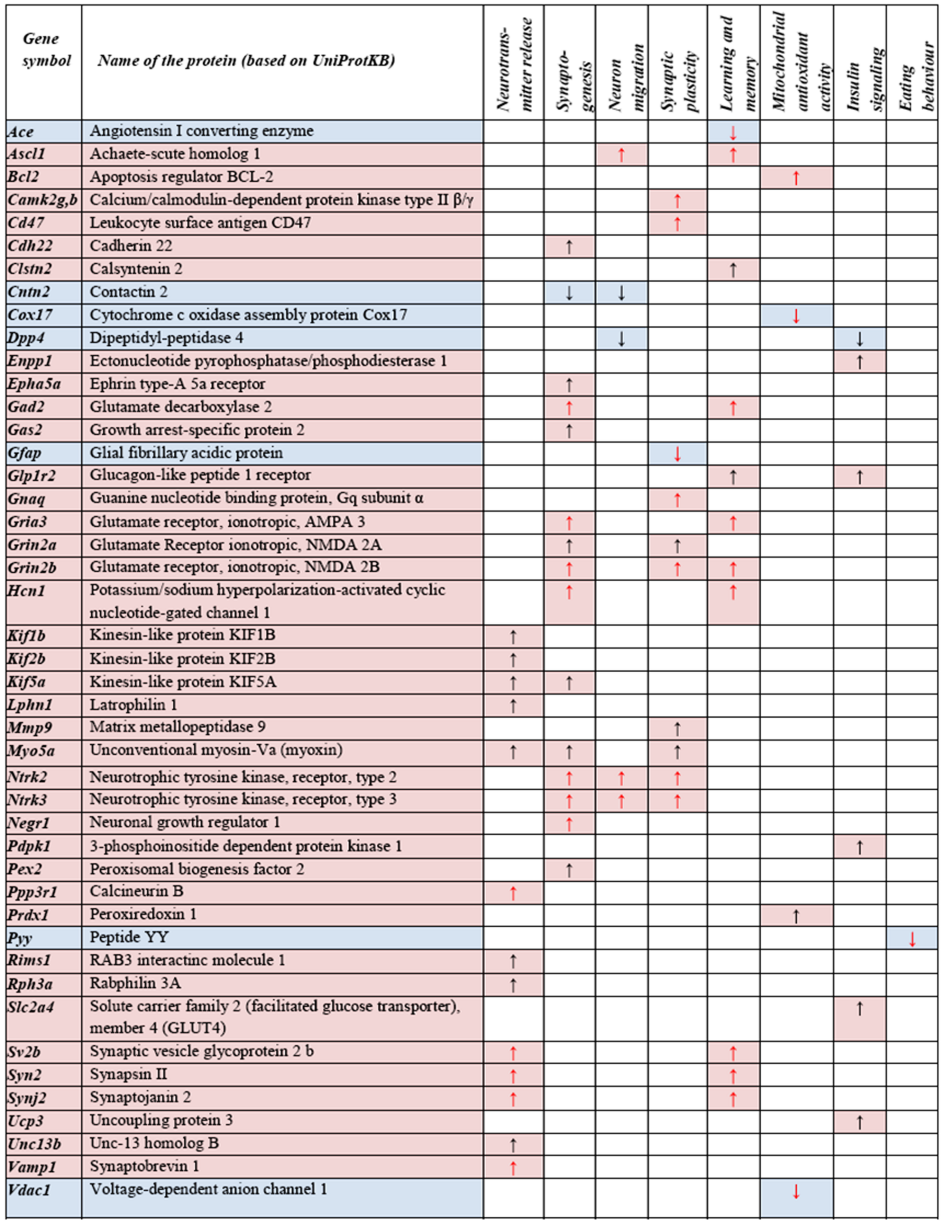
List of significantly changed genes with a potential/proven role in depression or in antidepressant effect. Differentially expressed genes were selected from network analysis based on scientific knowledge and literature (red square: upregulated; blue square: downregulated genes; red arrows: genes have experimentally proven role in depression/antidepression; black arrows: candidate genes which based on other and this experiments have a high probability to play a role in antidepressant response, p<0.05).

From genes showed altered expression, 23 have been previously associated with depression or antidepressant therapy in published studies (Figure 3, red arrows). From these, 5 genes were downregulated (*Ace, Cox17, Gfap, Pyy, Vdac1*), while the others were upregulated (*Ascl1, Bcl2, Camk2b, Camk2g, Cd47, Gad2, Gnaq, Gria3, Grin2b Hcn1, Negr1, Ntrk2, Ntrk3, Ppp3r1, Sv2b, Syn2, Synj2, Vamp1*) (Figure 3).

#### (ii) Genes with suspected role in depression (candidate genes, based on previous studies or their function in CNS)

From both the microarray data and the network analysis, 23 genes have been selected, which, based on prior knowledge, may play role in the pathophysiology of depression or in the molecular mechanisms of antidepressant therapy (Figure 3, black arrows). From these 23 candidates, 2 were downregulated (*Cntn2, Dpp4*) and 21 were upregulated (*Camk2b, Cdh22, Clstn2, Enpp1, Epha5a, Gas2, Glp1r2, Grin2a, Kif1b, Kif2b, Kif5a, Lphn1, Mmp9, Myo5a, Pdpk1, Pex2, Prdx1, Rims1, Rph3a, Slc2a4, Ucp3, Unc13b*) (Figure 3).

## Discussion

In this study we describe transcriptomic changes in the frontal cortex of Dark Agouti rats after a 3-week-long VLX treatment. The dose of VLX was 40 mg/kg/day, which is sufficient to block the reuptake of both 5-HT and NA (smaller than 40 mg/kg/day doses of VLX block exclusively the reuptake of 5-HT [41]). Our results suggest that chronic VLX administration has its major effects on neurotransmitter release via the regulation of genes involved in vesicular exocytosis and receptor endocytosis. Simultaneously, VLX increases expressions of gene sets related to neuroplasticity, axonogenesis and cognitive function. Interestingly, VLX changes the expression of genes involved in antioxidant activity of mitochondria and also modulates genes related to the insulin signaling pathway. Although the networks clearly show the effect of VLX, interpretation of changes on gene level is also important, since GSEA is a method merely based on rank tests and misses sensitivity at the level of differential expression.

### Neurotransmitter release

Our results show that VLX modulates numerous genes involved in synaptic vesicular transport. There are some neurotransmitter release-related genes that have been described in the pathomechanism of depression, for instance the *synj2* (Synaptojanin 2), a gene involved in membrane trafficking, which has decreased expression in the temporal cortex of patients with major depressive disorder [42]. Our results show that this gene is upregulated by a chronic VLX administration to rats.

Some genes could be linked to antidepressant effects, e.g. *Vamp1* (Synaptobrevin 1), a synaptic vesicle docking and/or fusion protein, the expression of which is increased in rat FC after chronic imipramine or sertraline treatment [43], [44]. Another example is *Syn2* (Synapsin II), a neuronal phosphoprotein that coats synaptic vesicles and regulates neurotransmitter release, and has been observed to be upregulated in prefrontal cortex of patients taking Lithium [45], or it is worth mentioning another vesicular protein, *Sv2b* (Synaptic vesicle glycoprotein 2b), which is upregulated after imipramine treatment in the frontal cortex of rats [44]. Like *Syn2* and *Sv2b* genes, *Ppp3r1* (Calcineurin B), the regulatory subunit of calcineurin, also showed increased expression after VLX treatment in our study. Notably, calcineurin interacts with the serotonin transporter modulating its plasma membrane expression and serotonin uptake [46]. Moreover, calcineurin has also direct antidepressant-like effects. In the study of Yu and coworkers, inhibition of calcineurin in the medial prefrontal cortex of rats induced depressive-like behaviour through mTOR signaling pathway [10].

VLX significantly increases the expression of other vesicle-related genes, the role of which in depression or antidepressant therapy has not been proven yet, although, based on their physiological function and altered expression levels after VLX, one can speculate on their potential involvement in these processes. Such genes comprise the kinesin-family member proteins (*Kif1b, Kif2b* and *Kif5a*; Kinesin family member 1B, 2b and 5a), which are involved in the neuronal transport of organelles, synaptic vesicle precursors, neurotransmitter receptors, cell signaling molecules, cell adhesion molecules and mRNAs in the nervous system (Figure 3) [47]–[49]. VLX also increases the mRNA levels of *Myo5a* (Myosin VA), a myosin V heavy-chain gene, being involved in the cytoplasmic vesicle transport along actin filaments [50], together with *Unc13b* that is required for priming synaptic vesicles for exocytosis [51]. Besides that, VLX up-regulates *Rims1* (RAB3 interacting molecule 1), *Rph3a* (Rabphilin 3A) and *Lphn1* (Latrophilin 1), which genes play a role in the regulation of synaptic vesicle exocytosis and neurotransmitter release in neurons [52]–[54].

### Synaptogenesis and neuron migration

Synaptogenesis declines in MDD, thus its reversal by chronic antidepressant treatment may provide a promising new direction in pharmacotherapy. Network hypothesis suggests that antidepressants reactivate or promote a juvenile-like state of brain plasticity and changes the strength of existing synapses. This facilitates the reorganization of cortical networks for a better environmental adaptation [3]. Our results show, that after VLX treatment, gene sets related to synaptogenesis or neuronal rearrangement were changed such as “neuronal death (NES = 1.5)”or “neuron migration (NES = 1.716)” (Figure 2B).

One upregulated gene (*Negr1*; Neurolnal growth regulator 1) in this group has been found to be increased at protein level also in the cerebrospinal fluid of depressive patients [55]. The question, whether it is part of the antidepressant effect of VLX or not, is hard to decide regarding the contradiction between our findings and the measurements from cerebrospinal fluid. However, there is growing literature about compartment-selective expression of genes in the central nervous system, which state that each brain region or even each neuron possesses a unique transcriptomic pattern and could react to environmental influences differently [56]. Since the major function of this gene is to promote axon regeneration, elevation of Negr1 in the FC could be part of the effect of VLX [57].

The gene sets showing significant alterations include many upregulated genes that could have potential role in antidepressant effect: cadherins, such as *Cdh22* (Cadherin 22), which play a role during migratory and lamination processes as well as axon guidance in temporal cortex of mice [58]; *Eph5a* (Ephrin 5a receptor), which is involved in the proper assembly of local cortical columns in rat developmental cortex [59]; *Gas2* (Growth arrest-specific protein 2), which is assumed to be involved in the maintenance of the subventricular stem cell niche and neuron apoptosis [60]. VLX also upregulated *Pex2* (Peroxisomal biogenesis factor 2). Mutant form of Pex2 is responsible for abnormal neuronal migration in Zellweger syndrome (peroxisome biogenesis disorder) [61]. VLX administration downregulates TAG-1 (Cntn2; Contactin 2), a member of the immunoglobulin superfamily. This gene product is present on corticofugal fibers and serves as a substrate for the migration of GABAergic interneuron. Blocking TAG-1 function in mouse cortical slices with anti-TAG-1 antibodies results in a marked reduction of migrating GABAergic interneurons [62], [63]. In this context, VLX acts as an inhibitor rather than an activator of neuronal migration.

### Synaptic plasticity

Synaptic pathology has received increasing interest as a key feature of mood disorders [9]. In network B (Figure 2/B), several functional categories are provided, which suggest the effect of VLX on synaptic plasticity in the FC, such as “Regulation of synaptic plasticity (NES = 1.79)”, “Synapse organization (NES = 1.59)”, “Neuron-neuron synaptic transmission (NES = 1.71)” and “Neuron projection terminus (NES = 1.67)” (Figure 2B).

We also found increase in the expression of Trk genes after chronic VLX administration (Figure 3). Trk genes (*Ntrk2*; Neurotrophic tyrosine kinase, receptor type *2, Ntrk3*; Neurotrophic tyrosine kinase, receptor type3) encode tyrosine kinase transmembrane receptors that are stimulated by neurotrophins such as BDNF (Brain Derived Neurotrophic Factor), NT-3 (Neurothrophin-3) or NT-4 (Neurothorphin-4), and are responsible for the transduction of signals controlling neuropoesis and neuron survival in the central nervous system. Their functional polymorphism and declined expression in the FC has been associated with depression as it was shown by previous studies [9], [64].

Depressed patients often show abnormalities in glutamatergic neurotransmission [65], and in some cases, this is due to a polymorphism of the GRIA3 gene (Glutamate receptor, AMPA 3) [66]. VLX treatment upregulated GRIA3, which suggests glutamate-based effects in the mechanism of the drug and supports previous findings regarding the influence of SSRIs on the glutamatergic-system [67], [68]. Also, NMDA (N-methyl-D-Aspartate) receptors *Grin2a* (Glutamate receptor ionotropic, NMDA 2A) and *Grin2b* (Glutamate receptor ionotropic, NMDA 2B) play key role in the pathology of mood disorders and their polymorphisms could be associated with depression [69]. In our samples VLX upregulated both *Grin2a* and *Grin2b*. Overexpression of these genes could have beneficial function in depression, as these receptors have major role in the regulation of synaptic plasticity [70], [71].

Cunha and coworkers suggested that the *Camk2* genes (Calcium/calmodulin-dependent protein kinase II β/γ) may have important beneficial effects in the treatment of depressive disorders, since the activation of these genes has antidepressant-like effects [72]. Our results also show induction of *Camk2b* and *Camk2g* after VLX treatment providing additional evidence for this finding.

*Gnaq* (Guanine nucleotide binding protein, q polypeptide) proteins represent a family of heterotrimeric proteins that couple cell surface 7-transmembrane domain receptors to intracellular signaling pathways. As behavioural studies of Frederick and co-workers have indicated, signaling through Gnaq is necessary for spatial memory [73]. VLX also has memory improving effects in rats [74]. Additionally to the findings above, we found an elevated mRNA expression of Gnaq in the frontal cortex of rats, which suggests, that a G-protein-coupled second messenger signaling pathways may play an important role in memory related action of this drug.

*Cd 47* (CD47 antigen) protein is involved in the regulation of neuronal networks in complex with other proteins. Mice lacking *Cd47* protein manifested prolonged immobility (depression like behaviour) in the forced swim test [75], [76]. Our results show that VLX increased the activity of this gene in the FC of Dark Agouti rats.

There is growing evidence that *Mmp9* (Matrix metallopeptidase 9) gene, which is induced by VLX in our experiments, is involved in synaptic plasticity and cognitive processes. Studies with transgenic animals show that mice over-expressing *Mmp9* display enhanced performance in both the non-spatial novel object recognition and the spatial water-maze task [77]. Their enhanced performance could be explained by an increased dendritic spine density observed in the hippocampus and cortex following behavioural testing [77].

*Gfap* (Glial fibrillary peptide 1 receptor) gene codes the glial fibrillary acidic protein, an intermedier filament maintaining the shape and movement of astroglial cells [78] It is also postulated, based on post-mortem human studies, that reduction of *Gfap* expression in astrocytes of fronto-limbic brain regions is part of MDD pathology [79]–[81]. Unexpectedly, our results also show reduction in *Gfap* levels after VLX, which points to the need of further experiments to clarify the role of this gene in mood-related disorders.

### Behaviour, learning and memory

In our study there were many memory-associated networks showing significant upregulation, such as “Long-term synaptic potentiation (NES = 1.4)”, “Long-term memory (NES = 1.65)” or “Glutamate signaling pathway (NES = 1.699)” (Figure 2B).

Considering gene level, we found many genes, which were modulated by chronic VLX treatment in the FC (Figure 3). For example, it elevates *Gad2* (Glutamic acid decarboxilase 2), the rate limiting enzyme for the conversion of glutamic acid to gamma-aminobutyric acid (GABA). There are no previous studies on Gad2 expression in FC in depression, but in the cingulate cortex of postmortem human subjects with MDD, a significant reduction in *Gad2* expression leading to GABA depletion has been demonstrated [82].

Since *Grin2b* is involved in long-term potentiation and there is an association between *Grin2b* single nucleotide polymorphisms (SNPs) and MDD [69], it may provide evidences for the role of this gene in memory loss of patients with depression. Upregulation of this gene by chronic VLX treatment underlines the positive effects of this antidepressant on memory loss during depression.

The observation that captopril induced an antidepressant effect in hypertensive patients [83] led to the suggestion that the brain renin-angiotensin system (RAS) may be involved in depression, and inhibition of the RAS may have antidepressant effect. On the other hand, the angiotensin-converting enzyme (*Ace*) besides converting angiotensin I to angiotensin II, is also involved in the degradation of neuropeptides, such as substance P, and elevation of this neuropeptide in the brain causes depression-like symptoms [84]. These contradictory findings, with our results demonstrating diminished *Ace* levels after VLX treatment, at least in part, support the fact that antidepressants exert their positive effects by inhibiting the brain RAS.

Studies indicate that various SNPs which are associated with lower expression of *Clstn2* gene (calsyntenin 2; cadherin type protein) can worsen episodic memory performance [85]. VLX treatment increased the expression of Clstn2, supporting its possible beneficial effects on memory.

HCN1 (Hyperpolarisation-activated cyclic nucleotide gated potassium channel 1) protein, which controls the way how neurons respond to synaptic input, is also called “pacemaker protein”, as it has oscillatory activity [86]. It is assumed, that this gene is important in memory, since its deletion causes profound motor learning and memory deficits in swimming and rotarod tasks [87]. In our experiment, chronic VLX upregulated *Hcn1* in the FC, that could also have importance in memory performance.

Although *Hcn1* upregulation in FC could be associated with a better memory performance, it is also known, that reduction of *Hcn1* in the dorsal hippocampal CA1 region produces antidepressant-like effects in mice [88]. This could be another evidence for the fact why it is important to study gene expression in different brain areas separately.

There are many genes involved in synaptogenesis, synaptic plasticity and transmission, which change their expression levels after learning. One of them is *Ascl1* (Achaete-scute complex like 1) [89], [90], which shows an increased expression in prefrontal cortex and hippocampus (HC) as it has been studied in water maze spatial memory performance test. This gene is also increased in our experiments after chronic VLX treatment.

Another gene, with significantly altered expression is *Glp1r2* (Glucagone-like peptide 1 receptor), which binds to GLP1 and plays a significant role in the regulation of both appetite and the gut-brain-pancreatic axis [91]. *Glp1r2*-deficient mice have a phenotype characterized by a learning deficit, which is restored after hippocampal *Glp1r2* gene transfer. In addition, rats overexpressing *Glp1r2* in the HC show improved learning and memory [91]. Although we studied changes in FC and not in the HC, increased expression of *Glp1r*2 in this brain region after VLX could also be important in memory related processes.

### Mitochondrial antioxidant activity

Mitochondrial function has an important role in the pathomechanism of depression. Studies on post-mortem tissues from human subjects have shown that the activity of mitochondrial complex I is decreased, while the oxidative damage is increased in the prefrontal cortex of patients with MDD [92]. Unexpectedly, VLX treatment decreased the expression of one member of the terminal mitochondrial respiratory chain complex IV, the copper chaperone (*Cox17*) and also *Vdac1* (Voltage-dependent anion channel 1), a mitochondrial outer membrane protein [93], which does not support the hypothesis, that VLX has beneficial effects on mitochondrial respiratory function (Figure 3). On the contrary, VLX induced antiapoptotic (*Bcl-2*; B-cell CLL/lymphoma 2) and antioxidant (*Prdx1*; Peroxiredoxin 1 [94]) mitochondrial genes, which underlines its stimulating effects on some mitochondrial functions. Studies on post-mortem FC tissues from patients with bipolar disorder show that *Bcl-2* is downregulated in depression [95], and also a rat study suggests that in chronic mild stress, VLX reverses the activated pro-apoptotic pathways [96]. A previous study also shows that in mononuclear cells of lithium responder depressive patients, lithium treatment increases the expression of the anti-apoptotic gene *Bcl-2* [9]. *Bcl-2* overexpression could be related to the lithium protection against neuronal apoptosis and oxidative stress.

Interestingly, analyzing functional gene sets, all of them were downregulated while none of them showed upregulation after VLX (Figure 2/D).

### Insulin signaling

Individuals with depression have a higher risk of developing type II diabetes. Conversely, individuals with diabetes are at an elevated risk of developing depression. It is also known that there is a higher risk for cognitive impairment when insulin regulation is disrupted [97]. In our experiments, VLX increased gene sets related to insulin, such as “insulin receptor binding (NES = 1.55)”or “G1 S transition mitotic cell cycle (NES = 1.49)” (Figure 2C). Also on gene level, the mRNA levels of several genes related to insulin signaling are reduced after VLX treatment (Figure 3). For instance, a high-fat diet leads to insulin resistance causing the reduced level of serine exopeptidase (*Dpp4*, Dipeptidyl-peptidase 4), which is known to leaven neuronal insulin receptor function, brain mitochondrial function and cognitive function in rats [98].

Insulin treatment increases the synthesis of *Pdpk1* (3-phosphoinositide dependent protein kinase 1) [99] an inducer of PSD-95 protein, which is an adapter molecule of ion channel and neurotransmitter receptor clusters at the postsynaptic membrane of hippocampal neurons resulting in a long-lasting enhancement of receptor-mediated synaptic transmission [100]. Since the expression of *Pdpk1* is increased after VLX treatment, similar functional enhancement could be assumed.

Other insulin signaling-related genes were also up-regulated by VLX, such as (i) *Enpp1* (Ectonucleotide pyrophosphatase/phosphodiesterase1) [101], which modulates insulin sensitivity; (ii) *Slc2a4* (Facilitaded glucose transporter, GLUT4), which has cytoplasmic expression in the neurons, but hormones (insulin or leptin) could translocate it to the plasma membrane [102], [103]; (iii) *Ucp3* (Uncoupling protein 3), which prevents glucose-induced transient mitochondrial membrane hyperpolarisation, reactive oxygen species formation, and induction of apoptosis as it has been proven in dorsal root ganglion neurons [104]; (iv) *Glp1r2* (Glucagone-like peptide 1 receptor), which delays gastric emptying and regulate appetite [105].

### Comparison of different antidepressants and limitations of available data

SSRIs are widely studied in gene expression studies [106], but SNRI data are scarce, and are completely missing at a time point relevant for clinical studies or our data. For SSRIs the identified genes harbouring SNPs interacting with SSRI effects (or those showing changes in mRNA expression after SSRI treatment) show a wide variety from one SSRI to the other [106]. For example, 18 neuroplasticity genes were identified that interact with fluoxetine, but only 4 in the case of sertraline [106]. Some discrepancies could be explained by differences in receptor binding profile of the drugs. For example, fluoxetine is able to block GluN1/GluN2B receptors, and this effect may well have an action on excitotoxicity [107]. Our VLX data identify several new genes and pathways that were not depicted after any of the SSRIs. Furthermore, gene-gene interactions at the receptor level, e.g., *SLC6A4* and *CNR1*, or at the signal transduction level, e.g., Gi and Gq coupled pathways, may even strengthen the otherwise weak genetic effects in certain patient groups leading to the concept of personalized medicine, but these interactions could not be identified by simple approaches [108], [109]. The use of different parts of the cortex in transcriptomic studies could be another source of discrepancies. One week treatment with VLX causes activation in the frontal cortex, but opposite effects in the parietal cortex in an MRI study [17]. Thus, further complex human and clinically relevant rodent studies and review papers focusing on specific questions are needed [106], [108].

## Conclusions

**Figure 4:**
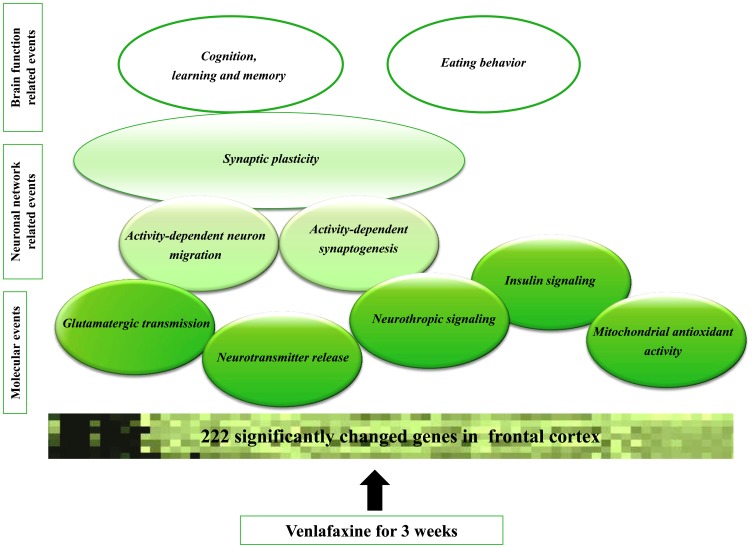
Summary of the transcriptomic changes in frontal cortex caused by three weeks long venlafaxine administration. Chronic VLX administration had major effect on neurotransmitter release, neurotrophic signaling, glutamatergic transmission, and also it influenced mitochondrial function and insulin signaling. These primary molecular mechanisms influence synaptogenesis, synaptic plasticity and finally lead to alteration in memory/cognition processes and eating behaviour.

In summary, considering expression patterns of genes and groups of genes following chronic VLX treatment in the FC of Dark Agouti rats, we identified several individual genes or gene networks that may contribute to changes in brain function and antidepressant properties of VLX (Figure 4). We demonstrated altered expression of genes involved in neurotransmitter release, neurotrophic signaling, glutamatergic transmission, as well as mitochondrial function and insulin signaling. The latter has not been investigated in depression so far. Upregulation of gene sets and genes relating to synaptic plasticity, cognition and memory after chronic VLX treatment is in correspondence with the synaptic (network) hypothesis of depression [9]. Since the mentioned, transcriptomic changes affect the frontal cortex and this brain region is known to be involved in the initiation of movements and motor coordination we assume that these changes could also explain the fact that venlafaxine improves cortical motor excitability. For example, Li and co-workers reported that VLX improved motor tasks and increased reaction speed in non-depressed persons [17]. Also, other studies show that SNRIs in rats or mice could affect locomotion and they have potential for ameliorating motor abnormalities [110], [111]. All these changes after 3 week long VLX treatment could be part of an adaptive response of frontal cortical neuronal networks.
