# Correction: Transcriptional Evidence for the Role of Chronic Venlafaxine Treatment in Neurotrophic Signaling and Neuroplasticity Including also Glutatmatergic- and Insulin-Mediated Neuronal Processes

**DOI:** 10.1371/journal.pone.0123269

**Published:** 2015-03-30

**Authors:** The PLOS ONE Staff

There is an error in the title of this paper: “Glutatmatergic” should be “Glutamatergic.” The correct title is: Transcriptional Evidence for the Role of Chronic Venlafaxine Treatment in Neurotrophic Signaling and Neuroplasticity Including also Glutamatergic- and Insulin-Mediated Neuronal Processes.

The correct citation is: Tamási V, Petschner P, Adori C, Kirilly E, Ando RD, Tothfalusi L, et al. (2014) Transcriptional Evidence for the Role of Chronic Venlafaxine Treatment in Neurotrophic Signaling and Neuroplasticity Including also Glutamatergic- and Insulin-Mediated Neuronal Processes. PLoS ONE 9(11): e113662. doi:10.1371/journal.pone.0113662
